# NeuroSeg-II: A deep learning approach for generalized neuron segmentation in two-photon Ca^2+^ imaging

**DOI:** 10.3389/fncel.2023.1127847

**Published:** 2023-04-06

**Authors:** Zhehao Xu, Yukun Wu, Jiangheng Guan, Shanshan Liang, Junxia Pan, Meng Wang, Qianshuo Hu, Hongbo Jia, Xiaowei Chen, Xiang Liao

**Affiliations:** ^1^Advanced Institute for Brain and Intelligence, Medical College, Guangxi University, Nanning, China; ^2^Department of Neurosurgery, The General Hospital of Chinese PLA Central Theater Command, Wuhan, China; ^3^Brain Research Center and State Key Laboratory of Trauma, Burns, and Combined Injury, Third Military Medical University, Chongqing, China; ^4^Center for Neurointelligence, School of Medicine, Chongqing University, Chongqing, China; ^5^School of Artificial Intelligence, Chongqing University of Technology, Chongqing, China; ^6^Brain Research Instrument Innovation Center, Suzhou Institute of Biomedical Engineering and Technology, Chinese Academy of Sciences, Suzhou, Jiangsu, China; ^7^Guangyang Bay Laboratory, Chongqing Institute for Brain and Intelligence, Chongqing, China

**Keywords:** two-photon Ca^2+^ imaging, generalized neuron segmentation, deep learning, attention mechanism, hybrid training

## Abstract

The development of two-photon microscopy and Ca^2+^ indicators has enabled the recording of multiscale neuronal activities *in vivo* and thus advanced the understanding of brain functions. However, it is challenging to perform automatic, accurate, and generalized neuron segmentation when processing a large amount of imaging data. Here, we propose a novel deep-learning-based neural network, termed as NeuroSeg-II, to conduct automatic neuron segmentation for *in vivo* two-photon Ca^2+^ imaging data. This network architecture is based on Mask region-based convolutional neural network (R-CNN) but has enhancements of an attention mechanism and modified feature hierarchy modules. We added an attention mechanism module to focus the computation on neuron regions in imaging data. We also enhanced the feature hierarchy to extract feature information at diverse levels. To incorporate both spatial and temporal information in our data processing, we fused the images from average projection and correlation map extracting the temporal information of active neurons, and the integrated information was expressed as two-dimensional (2D) images. To achieve a generalized neuron segmentation, we conducted a hybrid learning strategy by training our model with imaging data from different labs, including multiscale data with different Ca^2+^ indicators. The results showed that our approach achieved promising segmentation performance across different imaging scales and Ca^2+^ indicators, even including the challenging data of large field-of-view mesoscopic images. By comparing state-of-the-art neuron segmentation methods for two-photon Ca^2+^ imaging data, we showed that our approach achieved the highest accuracy with a publicly available dataset. Thus, NeuroSeg-II enables good segmentation accuracy and a convenient training and testing process.

## 1. Introduction

The fast advances in two-photon microscopy (Helmchen and Denk, 2005; Grewe et al., 2010; Stringer et al., 2019) and various Ca^2+^ indicators (Akerboom et al., 2013; Chen et al., 2013; Dana et al., 2019) have enabled researchers to record individual neurons *in vivo* at a large scale and high speed. Experiments have been performed to study brain functions with activities from many neurons in targeted brain regions. Accurately segmenting neurons carrying biological information is an essential step for analyzing the spatiotemporal data recorded by functional imaging experiments. Manual neuron segmentation is accurate and can screen out regions with unnecessary information, and thus, the manual segmentation result is defined as ground truth (GT). However, owing to the increasing amount of data (Harris et al., 2016) generated by the large size of the imaging field and the number of recorded neurons (Kim and Schnitzer, 2022), human annotators encounter a considerable workload. In addition, different annotators have their specific neuron labeling criteria, which may generate inconsistent results.

In the last decade, neuron segmentation approaches have continuously advanced in accuracy and computational speed. Currently, the methods can complete processing with a speed far exceeding that of human annotators and provide segmentation accuracy that is close to that of human annotators (Pnevmatikakis, 2019; Soltanian-Zadeh et al., 2019). The neuron segmentation methods are currently divided into two categories: unsupervised and supervised methods. For the first category of neuron segmentation methods (unsupervised), they typically identify pixels representing a neuronal structure and integrate these pixels into a region of neurons by intensity. This type of algorithm normally segments neurons with component analysis, including principal component analysis or independent component analysis (PCA/ICA) (Mukamel et al., 2009), non-negative matrix factorization (NMF) (Maruyama et al., 2014) and constrained non-negative matrix factorization (CNMF) (Pnevmatikakis et al., 2016), or the activity model (Pachitariu et al., 2017). For example, Suite2p (Pachitariu et al., 2017) uses spatial region of interest (ROI) shapes and neuronal activity traces from imaging data to segment neurons.

For the second category of neuron segmentation methods (supervised), they are trained to extract neuron features from labeled two-dimensional (2D) data (images) or three-dimensional (3D) data (videos). Convolutional neural networks (CNNs) are typically designed for supervised learning. Based on different types of data processing, CNNs can be divided into 2D CNN and 3D CNN. 2D CNN extracts features with training on manually labeled masks in image. For example, Mask region-based convolutional neural network (R-CNN) (He et al., 2017) is an instance segmentation algorithm and it can be applied for segmenting neuron in an image. In contrast to 2D CNN, 3D CNN is trained to extract features from labeled video data. For example, STNeuroNet (Soltanian-Zadeh et al., 2019) was proposed to use a 3D CNN for neuron segmentation and exploit the spatiotemporal information in two-photon Ca^2+^ imaging data. Shallow U-Net Neuron Segmentation (SUNS) (Bao et al., 2021) uses shallow CNN with U-shaped architecture to extract the spatial features of neurons, realizing fast and accurate segmentation. The 2D CNN and 3D CNN have their specific advantages and limitations. Neuron segmentation algorithms with 2D CNN are flexible and fast (Stoyanov et al., 2018). For this class of methods, the images of the training dataset can cover various Ca^2+^ indicators, imaging scales and imaging depths, which help this class of methods achieve a certain extent of generalizability and robustness. However, the temporal information of imaging data is lost when the video data are converted into an image. By contrast, neuron segmentation methods with 3D CNN can capture the temporal information of neurons (Bao et al., 2021), particularly they can help recognize overlapping neurons. However, this class of methods requires long recording and highly active neurons. In addition, some recent methods, e.g., CaImAn (Giovannucci et al., 2019), combine these two kinds of machine learning algorithms by identifying activity components using unsupervised learning and evaluating these components using supervised learning. However, it is still challenging for the existing methods to perform generalized neuron segmentation with complex two-photon Ca^2+^ imaging data, so we aim to develop a method that can accurately segment neurons in various situations.

In our previous study (Guan et al., 2018; Shen et al., 2018), NeuroSeg was developed to achieve unsupervised neuron segmentation for *in vivo* two-photon Ca^2+^ imaging data by using a generalized Laplacian of Gaussian filter to detect neurons and weighting-based segmentation to separate individual neurons. However, its model has the limitation of performing neuron segmentation in two-photon Ca^2+^ imaging data of different Ca^2+^ indicators. Hence this method demands further development. To segment both active and inactive neurons in imaging data across Ca^2+^ indicators, imaging scales, brain regions and imaging depths, here we propose NeuroSeg-II, a deep learning model based on an attention mechanism and enhanced feature hierarchy, to perform neuron segmentation in two-photon Ca^2+^ imaging with a 2D image processing approach. As the sparsely firing neurons may be hardly visible in the average or maximum projected images, the correlation map can make these neurons visible (Pachitariu et al., 2017). In preprocessing, we fused the average image with correlation map to integrate the spatial and temporal information and generate a new 2D image for neuron segmentation. To train and validate NeuroSeg-II’s performance, we used the datasets acquired from our lab and publicly available datasets. The results show that NeuroSeg-II solved the problem of generalized neuron segmentation in two-photon Ca^2+^ imaging data and achieved good performance across different Ca^2+^ indicators (OGB-1, Cal-520, and GCaMP6), multiple imaging scales, different brain regions and imaging depths. NeuroSeg-II used spatiotemporal activity information with fused images and successfully segmented active and inactive neurons, indicating that it has good generalizability for processing different types of imaging data. By comparing the other methods for neuron segmentation with the publicly available two-photon Ca^2+^ imaging dataset, we found that our approach outperformed other competitors in accuracy. Therefore, our deep learning approach is efficient in performing generalized neuron segmentation in two-photon Ca^2+^ imaging, which is complementary to NeuroSeg and may facilitate future neuroscience research.

## 2. Materials and methods

### 2.1. Functional two-photon imaging datasets

#### 2.1.1. Data acquisition in our lab

In this study, C57BL/6J mice (2–3 months old) were used for two-photon Ca^2+^ imaging experiments. The mice were provided by the Laboratory Animal Center at the Third Military Medical University, and the experimental procedures were performed based on protocols approved by the Third Military Medical University Animal Care and Use Committee.

The two-photon Ca^2+^ imaging experiments were conducted in the mouse auditory cortex (Li et al., 2017; Wang M. et al., 2020). After we anesthetized the mouse with isoflurane, the mouse’s skull was glued with a prefabricated plastic chamber. The auditory cortex region was exposed by a small craniotomy (∼4 mm^2^) and injected with indicator (OGB-1 AM, Cal-520 AM, or GCaMP6f). After 2 h, Ca^2+^ imaging was performed with a mode-locked Ti:Sa laser (Mai-Tai DeepSee, Spectra Physics, Santa Clara, CA, USA) delivering two-photon excitation light. A custom-built two-photon microscope system (LotosScan, Suzhou Institute of Biomedical Engineering and Technology, Suzhou, China) was used to record the imaging data (Jia et al., 2010, 2014).

This dataset consisted of 193 two-photon Ca^2+^ imaging videos, comprising 61 data samples for expressing the OGB-1 indicator, 127 data samples for expressing the Cal-520 indicator, and five data samples for expressing the GCaMP6f indicator. Three experienced annotators labeled each neuron independently and then compared their labeled results to produce a final consensus as the GT.

#### 2.1.2. Allen brain observatory (ABO) dataset

The ABO dataset consists of neuronal population imaging across different brain regions and layers with two-photon microscopy. We used the ABO dataset consisting of 132 images from ALLEN BRAIN ATLAS (Sessions A–C). This image dataset includes six images recorded at a depth of 175 μm in the rostrolateral visual cortex (VISrl), 12 images recorded at a depth of 175 μm in the posterolateral visual cortex (VISpm), nine images recorded at a depth of 275 μm in the VISpm, 16 images recorded at a depth of 175 μm in the VISp, 25 images recorded at a depth of 275 μm in the VISp, 12 images recorded at a depth of 175 μm in the lateral visual cortex (VISl), 15 images recorded at a depth of 275 μm in the VISl, six images recorded at a depth of 175 μm in the anteromedial visual cortex (VISam), 13 images recorded at a depth of 175 μm in the anterolateral visual cortex (VISal), and 18 images recorded at a depth of 275 μm in the VISal. All mice in the above experiments expressed the GCaMP6f indicator. Three experienced annotators labeled each neuron independently and then compared their labeled results to produce a final consensus as GT.

#### 2.1.3. Neurofinder challenge dataset

The Neurofinder dataset consists of neuronal population imaging across different brain regions with two-photon microscopy. The dataset was annotated in three different laboratories, resulting in diverse sub-datasets. We used 10 imaging data (videos) samples from this dataset. All mice expressed the GCaMP6s indicator. We used the neuron GT from the work of STNeuroNet (Soltanian-Zadeh et al., 2019). Each group of videos contained one training data sample and one testing data sample. To increase the number of images and improve image quality, we used the preprocessing method to convert each video dataset into seven corresponding images (six images from an evenly divided video and one image from the whole video).

#### 2.1.4. Large-field mesoscopic two-photon imaging dataset

A single image data sample of mesoscopic large-field imaging was recorded with a mouse expressing GCaMP6s under the thy-1 promoter (Sofroniew et al., 2016). As the dimensions of the image data are too large (1,792 pixels × 1,682 pixels) to fit the neural network, the image data cannot be tested directly. Thus, we segmented the original image into small images for testing. The neuron GT is provided with this dataset.

### 2.2. Image preprocessing

**FIGURE 1:**
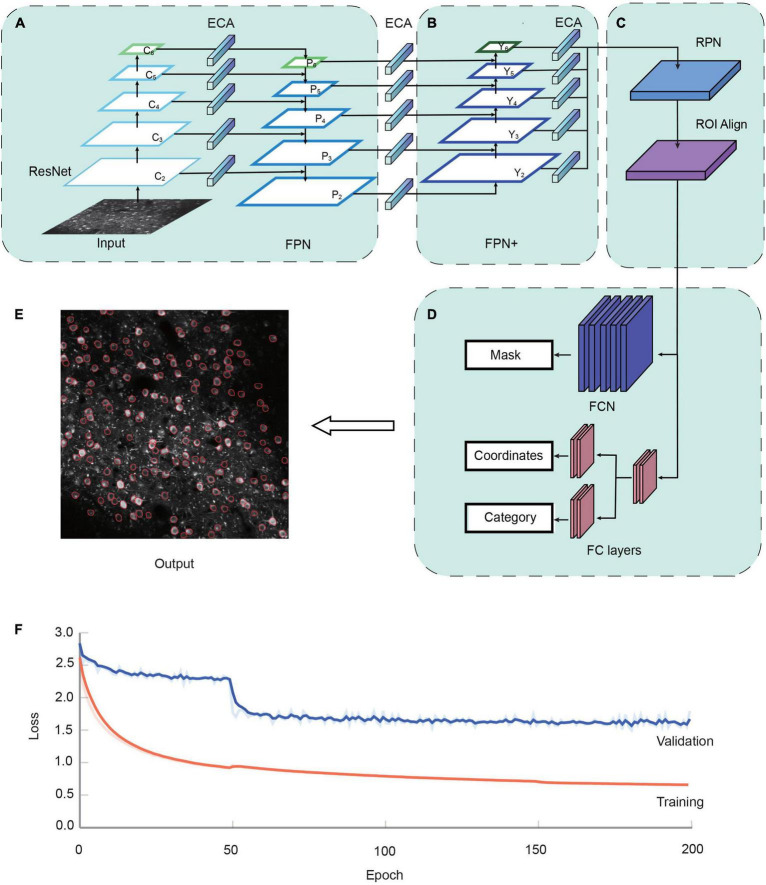
The NeuroSeg-II architecture. **(A)** The two-photon Ca^2+^ image is input to the network. ResNet uses the down-sampling structure of the backbone, and we added one more down-sampling layer after the C5 layer. Feature pyramid network (FPN) uses each layer of the ResNet output feature to the input corresponding to the up-sampling structure. The channel attention mechanism module efficient channel attention (ECA) was added to lateral connections. **(B)** FPN+ uses the additional down-sampling structure to obtain input from FPN. The channel attention mechanism module ECA was added between lateral connections. **(C)** Region selection and feature aggregation subnetwork. Region proposal network (RPN) uses the FPN to extract the feature map, score the front and back scenes, and select the target region. Region of interest (ROI) Align matches the original image with the feature image for feature aggregation. **(D)** Head network consists of a parallel mask segmentation branch and a classification/regression branch. **(E)** Neuron segmentation result by NeuroSeg-II [data from allen brain observatory (ABO) dataset, experiment ID: 511510945]. **(F)** The convergence of the proposed network in the training and validation datasets is demonstrated over the course of 200 epochs using TensorBoard. Loss values were normalized to better visualize trends. Training dataset, orange; validation dataset, blue.

First, the motion corrected imaging data (videos) were converted into images by average projection (Stoyanov et al., 2018) and correlation map (Foroosh et al., 2002; Alba et al., 2015) to represent the spatiotemporal information of neurons. To obtain the correlation map, we calculated the multidimensional correlation of each pixel and its surrounding pixels to localize the neurons. Here, we calculated a weighted multidimensional correlation (Pachitariu et al., 2017) as


(1) $$c_{w}\,\left(f_{1},f_{2},\cdots \right)=\frac{{||\sum_{i}a_{i}\,f_{i}||}^{2}}{\sum_{i}a_{i}\,{||f_{i}||}^{2}},$$

where *c_w_* is the calculation at each pixel for different dimensions, *f_i_* is the traces of neighboring pixels, and *a_i_* is a Gaussian kernel for weighting. The relatively large values of the correlation map indicate the neuron locations. We finally fused the average images with correlation maps to obtain new images. These new images were used as inputs to the network for training and testing, corresponding to the “Input” for NeuroSeg-II (Figure 1A).

### 2.3. Neural network architecture

The realization of neuron segmentation requires the accurate detection and segmentation of objects in the image. NeuroSeg-II is implemented with a combination of object detection and segmentation, with Mask R-CNN (He et al., 2017) being the backbone of our network model. To perform prediction for neurons in imaging data, the architecture uses ResNet (He et al., 2016) to extract features and the feature pyramid network (FPN) (Lin et al., 2017) as the feature hierarchy within the network (Figure 1A). FPN is an important network component for detecting objects at different scales. FPN is taking the advantages of both strong semantic information from the top layers and high-resolution information from the bottom layers. This approach allows the network to have good semantic and high-resolution information at different scales and enhances the performance of object segmentation. Based on this advantage, FPN enables efficient neuron detection and segmentation at different scales.

**FIGURE 2:**
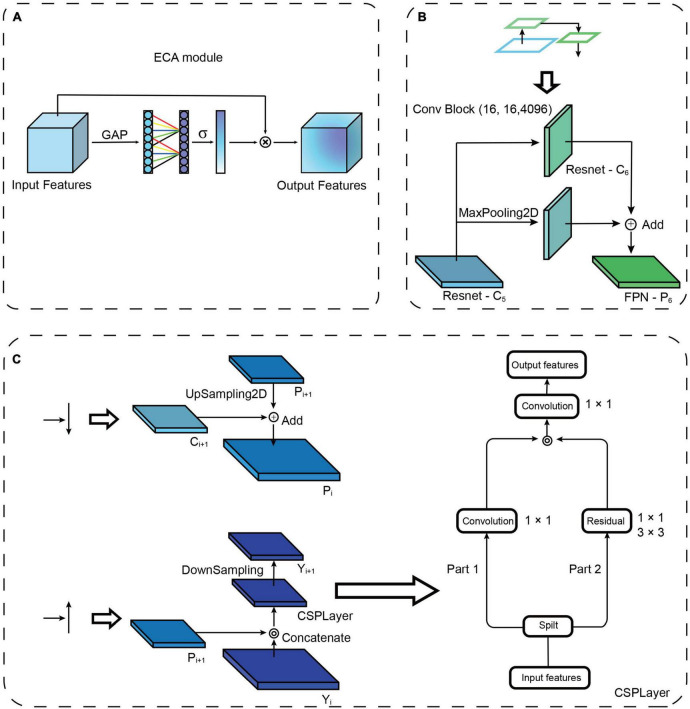
Attention mechanism and path augmentation modules. **(A)** Diagram of efficient channel attention (ECA) module. GAP, Global average pooling. ⊗: The output is combined with the input feature map. **(B)** Illustration of the structure of ResNet down-sampling path augmentation. **(C)** Left top: “Add” is used as the fusion method of up-sampling and lateral connection. Left bottom: “Concatenate” is used as the fusion method of down-sampling and the lateral connection. Right: Illustration of the structure of the CSPLayer.

As attention mechanisms have been reported to increase the power of emphasizing important objects and suppressing the background, we added an attention mechanism module to the lateral connection in the model to improve the feature extraction ability. Here, efficient channel attention (ECA) (Wang et al., 2019) is used to add lateral connections. The ECA consists of global average pooling (GAP) and fast one-dimensional (1D) convolutions (Figure 2A). The GAP is used to process the obtained aggregated features, and fast 1D convolution is used to generate channel weights. ECA is a modification of squeeze-and-excitation networks (Hu et al., 2018), which augment appropriate cross-channel interactions and eliminate dimensionality reduction to improve channel attention.

To propagate features with stronger semantics, we added a down-sampling path to shorten the information path between the top and bottom layers (Liu et al., 2018), and we enhanced the feature hierarchy to localize objects in the bottom layers (Figure 1A). Briefly, based on the four times down-sampling in ResNet, we added one down-sampling after ResNet-C5 (the last layer of ResNet), which changes the original four feature outputs into five feature outputs (Figure 2B). Following ResNet, the up-sampling and down-sampling paths also increase the input and output features, respectively. This modification reduces the loss of information and increases the utilization of feature information at diverse levels, thus enriching the feature information of small objects. To disseminate features in the network, two kinds of feature integration approaches are used (Figure 2C), “Add” (He et al., 2017) and “Concatenate” (Bochkovskiy et al., 2020), for each layer’s transverse connection paths. The “Add” increases the number of image features but does not increase the description image dimension. The “Concatenate” increases the features of the image, enriching the features of the image and reducing the redundancy of information (Huang et al., 2017). A cross-stage partial layer (CSPLayer) (Wang C.-Y. et al., 2020) was added after the “Concatenate” (Figure 2C), which strengthens the learning ability of the network. It also eliminates the computing bottleneck and reduces the memory cost of using “Concatenate” multiple times (Huang et al., 2017).

To further strengthen the object localization capability for the feature hierarchy, and combine the response of higher-level neurons to the whole of objects and the response of lower-level neurons to local textures, we added a new information path from the top to bottom layers connected to FPN, which we call “FPN+” (Figure 1B). The “FPN+” performs step-by-step down-sampling to generate new feature maps and obtain higher-resolution feature maps and coarser maps through lateral connections. Hence, the “FPN+” generates the new feature maps from Y_2_ to Y_6_. The input image was combined with the feature map to prepare for the subsequent image segmentation (Figure 1C). The following Head network was used to classify and segment the original image (Figure 1D) and generate the output result (Figure 1E). The loss function of network model combines the losses of classification, regression and segmentation mask. The region selection, feature aggregation, and Head network are the same as those in Mask R-CNN.

### 2.4. Data augmentation

To enhance the training effect, we used the imgaug tool to expand the training sample. In the training process, the original image was flipped horizontally (50% probability), flipped vertically (50% probability), rotated (90, 180, 270^°^), scaled (0.8–1.5 times), and added with Gaussian noise (intensity from 0.0 to 5.0). We randomly used zero to five items, as mentioned above, in the training network.

### 2.5. Model training strategies

#### 2.5.1. Model training with hybrid dataset

This training strategy was used to perform the model training and the performance testing of the attention mechanism module, the enhanced feature hierarchy, and the improvement from Mask R-CNN to NeuroSeg-II. The dataset from our lab and the ABO dataset were used, including 325 images (193 images from our lab and 132 images from the ABO dataset). It was divided into three sub-datasets: the training dataset (223 images), the validation dataset (51 images), and the testing dataset (51 images). Using a transfer learning approach, we used this hybrid dataset to train the ResNet based on the model parameters pre-trained on the ImageNet dataset. We trained NeuroSeg-II for 200 epochs. The training process was divided into three stages. The first stage froze all layers except the Head network and consisted of 50 epochs running at a learning rate of 1 × 10^–3^. The second stage thawed the global network and consisted of 100 epochs running at a learning rate of 2 × 10^–4^. The third stage reduced the learning rate to 1 × 10^–4^ and consisted of 50 epochs. Each epoch consisted of 500 steps with a batch size of two. By using the above strategy to train our network, it shows that the loss was decreased clearly at the initial learning stage (before 50 epochs), and was converged at the end of the learning process (Figure 1F).

#### 2.5.2. Model training with the neurofinder dataset

To compare the neuron segmentation performance of NeuroSeg-II with other methods, we used the Neurofinder dataset for evaluation. All the methods were trained and tested by two-round hybrid cross validation. Through the evaluation of the methods using video data for training and testing, we trained the models, including SUNS (Bao et al., 2021), STNeuroNet (Soltanian-Zadeh et al., 2019), and CaImAn (Giovannucci et al., 2019), or ROI classifiers in Suite2p (Pachitariu et al., 2017). Five groups of video datasets (01.00–04.01) were trained together to generate one model. We used the trained models to test the five corresponding video data (01.00.test–04.01.test) and obtained the result, that is, the second round of cross validation of the training dataset and testing dataset interchange. We obtained the testing result of all 10 groups of the Neurofinder dataset. For the evaluation of the methods used image data for training and testing (NeuroSeg-II), we replaced the training dataset with 35 images (five groups of video datasets with seven images per dataset) and the testing dataset with five images (five groups of video dataset with one global image per dataset). We obtained the testing result of all 10 groups of the Neurofinder dataset. All the above methods were optimized according to their papers (Pachitariu et al., 2017; Giovannucci et al., 2019; Soltanian-Zadeh et al., 2019; Bao et al., 2021). We used the model provided by CITE-On (Sità et al., 2022) for training and testing. To adapt to the CITE-On neuron detection function, we converted the annotated neuron edges into bounding boxes. The training process of NeuroSeg-II was as follows: The network model pre-trained with the hybrid dataset was trained with the Neurofinder dataset for a total of 150 epochs, including two stages. The first stage froze all layers except the Head network and consisted of 20 epochs running at a learning rate of 1 × 10^–3^, and the second stage thawed the global network and consisted of 130 epochs running at a learning rate of 1 × 10^–3^. Each epoch consisted of 50 steps with a batch size of two.

To verify the rationality of two-round hybrid cross validation, we also used a 10-round single cross-validation procedure for training and testing NeuroSeg-II. In the 10-round (one-to-one) cross-validation procedure, we used each of the 10 groups in the dataset as the training data only once. In each round of cross validation, we used seven preprocessed images (evenly divided part in video) as the training dataset and one image (whole video) as the test dataset (e.g., 01.00 training, 01.00.test test). NeuroSeg-II’s training process was the same as that of the two-round hybrid cross validation. Each epoch consisted of 20 steps with a batch size of two.

### 2.6. Evaluation metrics

To evaluate segmentation methods, we compared the results with the GT (Soltanian-Zadeh et al., 2019). We performed the evaluation with three metrics (i.e., precision, recall, and F1-score), defined as follows:


(2) $$\text{Recall}=\frac{N_{\text{TP}}}{N_{\text{GT}}},$$

(3) $$\text{Precision}=\frac{N_{\text{TP}}}{N_{\text{detected}}},$$

(4) $$\mathrm{F1}-\mathrm{score}=2\times \frac{\text{Recall}\;\times \;\text{Precision}}{\text{Recall}\;+\;\text{Precision}},$$

where *N*_TP_ is the number of true-positive neurons, *N*_GT_ is the number of manually labeled neurons, and *N*_detected_ is the number of detected neurons. The intersection-over-union (IoU) metric and the Hungarian algorithm were applied to calculate the degree of overlap between the detected neuron masks and the GT (Soltanian-Zadeh et al., 2019). The IoU was measured with two binary masks, *m_1_* and *m_2_*:


(5) $$\text{IoU}\,\left(m_{1},{\text{m}}_{2}\right)=\frac{\left|m_{1}\cap m_{2}\right|}{\left|m_{1}\cup m_{2}\right|}.$$

Then, the distance (Dist) between a pair of masks is measured as


$$\text{Dist}\left(m_{i}^{\text{GT}},M_{j}\right)$$

(6) $$=\;\left\{\begin{array}{cc} 1-\text{IoU}(m_{i}^{\text{GT}},M_{j}), & \text{IoU}(m_{i}^{\text{GT}},M_{j})\geq 0.5\\ 0, & m_{i}^{\text{GT}}\subseteq M_{j}\;\text{or}\;M_{j}\subseteq m_{i}^{\text{GT}}\\ \infty , & \text{otherwise} \end{array}\right.,$$

where $m_{i}^{\text{GT}}$ is the mask *i* for the GT, and *M_j_* is mask *j* for the detected neuron. After that, the Hungarian algorithm was used to generate the true-positive neuron masks.

### 2.7. Statistical analysis

In this study, all summary data were expressed as the mean ± SEM. For all statistical tests, a two-sided Wilcoxon signed-rank test was applied with MATLAB 2018b (MathWorks, USA) (*P < 0.05; ^**^P < 0.01; ^***^P < 0.001; and ns, not significant). The results were deemed statistically significant when *P* < 0.05. Statistical parameters, including the definitions and exact values of n, were reported in the text and figure legends. No data was considered an outlier and removed from statistical analyses.

## 3. Results

The data preprocessing, network model training, and testing were conducted using Ubuntu 20.04.4 LTS, Intel Xeon Gold 6152 CPU, 256 GB RAM, NVIDIA Tesla V100 GPU.

### 3.1. Image fusion to represent neuronal spatiotemporal activity

**FIGURE 3:**
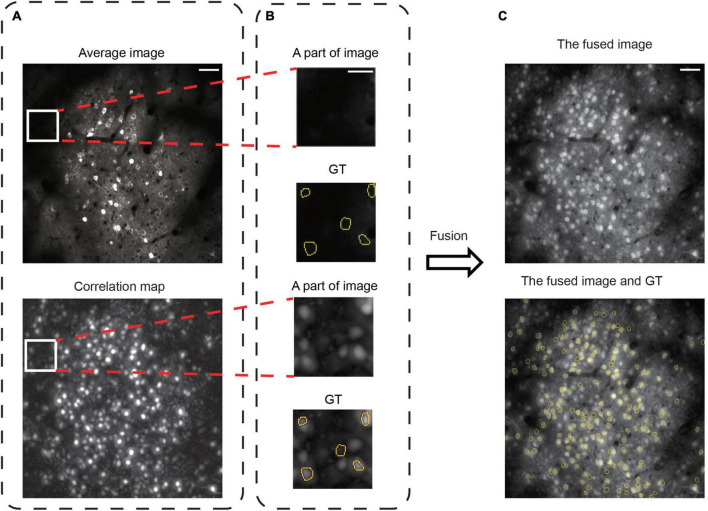
Image fusion to improve spatiotemporal information representation. **(A)** The images are the average image and correlation map over the recording of one dataset, 02.00 test, from Neurofinder data. Scale bars, 50 μm. **(B)** Some invisible, sparsely firing neurons are in the average image, and the correlation map can make these neurons visible. The GT is from STNeuroNet. The yellow outlines indicate the GT neurons. Scale bars, 20 μm. **(C)** The average image and the correlation map were fused and supplemented with the spatiotemporal information of the 2D image. The GT is from STNeuroNet. The yellow outlines indicate the GT neurons. Scale bars, 50 μm.

The 2D image generated from functional imaging data can represent the temporal information in the whole video by average projection (Stoyanov et al., 2018), maximum projection (Shen et al., 2018), or correlation map (Spaen et al., 2019; Bao et al., 2021). In the average image, the neurons often have a “donut” structure (Pachitariu et al., 2013; Apthorpe et al., 2016; Figure 3A) because Ca^2+^ indicators are normally expressed in the cytoplasm of a neuron (Chen et al., 2013). However, this criterion is insufficient because sparsely firing neurons are invisible (Figure 3B) in the average image (Stringer and Pachitariu, 2019). In contrast, they are visible in the correlation map of each pixel with its near pixels (Smith and Hausser, 2010; Portugues et al., 2014; Figures 3A, B). Moreover, the opposite is also true: Many neurons visible in the average image are invisible in the correlation map, suggesting that their fluorescence only reflects the baseline Ca^2+^ in these neurons (Stringer and Pachitariu, 2019). Hence, the previous neuron segmentation methods with 2D images had unsatisfactory recognition accuracy for overlapping and sparsely firing neurons (Soltanian-Zadeh et al., 2019). We propose image fusion as the preprocessing method to tackle this problem. We used image fusion as the preprocessing method to enhance the representation power in 2D image data. The fusion of the two kinds of images enriched the neuron spatial features in 2D images and recovered some lost temporal information in the average image (Figures 3A, C). Therefore, we used the preprocessed images as the training and testing dataset for NeuroSeg-II.

### 3.2. The attention mechanism and modified feature hierarchy improved the neuron segmentation performance

**FIGURE 4:**
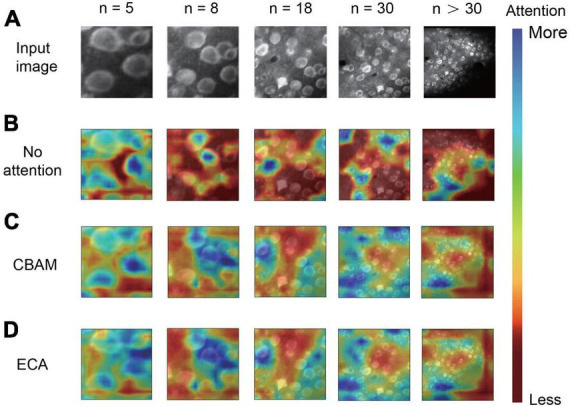
Visualization of attention mechanisms on different numbers of neurons. **(A)** The input two-photon images with different numbers of neurons. **(B–D)** The difference in image feature focusing between no attention module **(B)**, convolutional block attention module (CBAM) **(C)**, and efficient channel attention (ECA) **(D)**. We use Grad-CAM for the visualization of attention effects (n is the number of neurons in each image).

**FIGURE 5:**
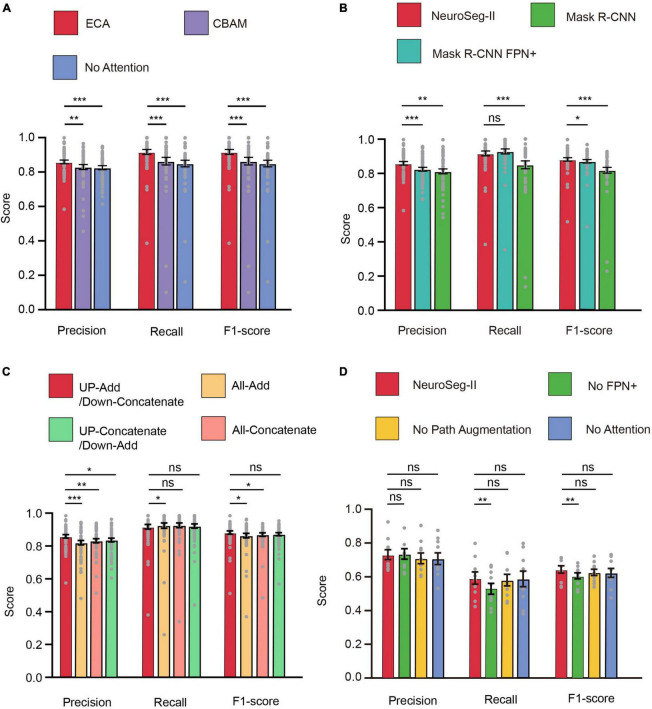
Attention mechanism, FPN+, and path augmentation increase neuron segmentation accuracy. **(A)** The efficient channel attention (ECA) module within our model was superior to other approaches for neuron segmentation (**P < 0.01, ***P < 0.001; *n* = 51 images; and error bars are SEM). **(B)** NeuroSeg-II’s neuron segmentation score was superior to those of Mask region-based convolutional neural network (R-CNN) and Mask R-CNN FPN+ (adding FPN+ to Mask R-CNN) (*P < 0.05, **P < 0.01, ***P < 0.001, and *n* = 51 images; ns, not significant; and error bars are SEM). The results were obtained using hybrid training. **(C)** The neuron segmentation score of using “Add” for up-sampling and “Concatenate” for down-sampling was superior to those of other methods (*P < 0.05, **P < 0.01, and ***P < 0.001; *n* = 51 images; ns, not significant; and error bars are SEM). The results were obtained using hybrid training. **(D)** The ablation study for testing the network components including attention module, path augmentation and “FPN+” (**P < 0.01; *n* = 10 images; ns, not significant; and error bars are SEM). The results were obtained using two-round hybrid cross validation with the Neurofinder dataset. All *P*-values were calculated with a two-sided Wilcoxon signed-rank test. The gray dots represent the scores for each testing image.

For the neuronal population imaging data, the uneven background can generate some neuron-like structure and thus affect the segmentation task (Figure 4A). To focus the neurons and exclude the background influence, we added an ECA-based attention mechanism module to the lateral connection process in our network model (Figure 1A). Here, each channel of the module plays the role of a feature detector to focus on significant parts of the input image. To observe the regions that are important for detecting neurons, we visualized how the attention module emphasizes features (Figure 4). The figure shows that the attention mechanism could concentrate on multiple objects instead of on a single object. We can also clearly see that the masks from ECA (Figure 4D) covered the neuron regions better than the method without using an attention mechanism (Figure 4B) for different numbers of neurons (*n* = 5, 8, 18, 30 and *n* > 30) in the field of view (FOV). That is, the ECA-integrated network learns well to exploit information in neuron regions and aggregate features from them. The observations confirm that the feature refinement process of ECA eventually leads networks to use the given features well. The method without using an attention mechanism only focuses on a few neurons, leading to a significant loss of object information. In contrast, unlike channel attention, the spatial attention module screens the input image location information. Hence, we compared ECA with the convolutional block attention module (CBAM) (Figure 4C; Woo et al., 2018) and the model without using an attention mechanism (Figure 5A). The CBAM uses a combination of channel attention and spatial attention mechanisms. The comparison results show that the ECA in NeuroSeg-II achieved a significantly higher F1-score than that of the other two methods (*P* < 0.001, two-sided Wilcoxon signed-rank test, *n* = 51 images). Hence, augmenting spatial attention mechanism did not screen the location information of neurons well, so ECA was better than CBAM and the method without using an attention mechanism for neuron segmentation.

In two-photon imaging experiments, the recorded neurons could be small and dense in a large FOV, so neurons contain too few discriminative features owing to the few pixels (Kisantal et al., 2019). A small object is defined as an object with pixel values of less than 32 × 32 (Bosquet et al., 2018), and the diameter of neurons from ABO and Neurofinder datasets being 10–30 pixel values. A previous study (Zeiler and Fergus, 2014) reported that higher-level objects were activated entirely. In contrast, lower-level objects were activated locally. This indicates that it is necessary to augment the top-down information path to propagate features and enhance feature extraction capability in FPN. To enhance feature extraction at multi-scales, we added “FPN+” and path augmentation to the feature hierarchy in the network model (Figures 1A, B).

To further validate the path augmentation effect, we compared NeuroSeg-II with Mask R-CNN and Mask R-CNN FPN+ (a modified version with adding “FPN+” to Mask R-CNN) for neuron segmentation (Figure 5B). The results demonstrate that the precision and F1-score of NeuroSeg-II were significantly higher than those of the other two methods (*P* < 0.05, two-sided Wilcoxon signed-rank test, *n* = 51 images). The recall rate of NeuroSeg-II (0.917 ± 0.014) was slightly lower than that of Mask R-CNN FPN+ (0.928 ± 0.014; *P* = 0.0665, two-sided Wilcoxon signed-rank test, *n* = 51 images) and significantly higher than that of Mask R-CNN (*P* < 0.001, two-sided Wilcoxon signed-rank test, *n* = 51 images).

Our network mode uses “Add” as the up-sampling and “Concatenate” as the down-sampling fusion approach (Figure 2C). To validate the performance of this integration approach, we compared it with the other three combination options (Figure 5C). The results show that the combination of “Add” as the up-sampling method and “Concatenate” as the down-sampling method had the highest performance to carry out feature integration. This method’s precision was significantly higher than that of the other three approaches (*P* < 0.05, two-sided Wilcoxon signed-rank test, *n* = 51 images). The recall rate (0.917 ± 0.014) was lower than those of the other three methods (0.924 ± 0.016, 0.926 ± 0.014, and 0.922 ± 0.013; *P* < 0.05, *P* = 0.1449, and *P* = 0.6879; two-sided Wilcoxon signed-rank test, *n* = 51 images). The F1-score (0.882 ± 0.010) was significantly higher than “All-Add” and “All-Concatenate” (0.863 ± 0.013 and 0.870 ± 0.011; *P* < 0.05, two-sided Wilcoxon signed rank test, *n* = 51 images), and higher than “Up-Concatenate/Down-Add” (0.872 ± 0.010; *P* = 0.1190, two-sided Wilcoxon signed-rank test, *n* = 51 images). The improvement of the effect of this combination method is due to richer image features during up-sampling that was used to increase the number of features by “Add.” Thus, the higher semantic information of features during down-sampling was used to enrich features by “Concatenate.”

In addition, we conducted ablation study to examine the effects of the attention mechanism and the modified feature hierarchy including path augmentation and “FPN+” (Figure 5D). The results show that all the network components affected precision and recall rate. In particular, the addition of FPN+ had an obvious effect on the recall rate. The precision of NeuroSeg-II (0.731 ± 0.029) was at the same level as that of ablating FPN+ (0.738 ± 0.030; *P* = 0.9219, two-sided Wilcoxon signed-rank test, *n* = 10 images) and was higher than other ablation results (attention ablation: 0.708 ± 0.033, *P* = 0.2754; path augmentation ablation: 0.710 ± 0.030, *P* = 0.0781; two-sided Wilcoxon signed-rank test, *n* = 10 images). The recall rate (0.591 ± 0.036) and F1-score (0.644 ± 0.022) of NeuroSeg-II were significantly higher than those of ablating FPN+ (*P* = 0.002 for both recall rate and F1-score, two-sided Wilcoxon signed-rank test, *n* = 10 images) and higher than other ablation results (recall rate: attention ablation, 0.586 ± 0.044, *P* = 0.6523; path augmentation ablation, 0.579 ± 0.032, *P* = 0.4258; F1-score: attention ablation, 0.624 ± 0.025, *P* = 0.1055; path augmentation ablation, 0.627 ± 0.017, *P* = 0.1055; two-sided Wilcoxon signed-rank test, *n* = 10 images). Therefore, the results indicate that the ablation of network components resulted in lower accuracy, all network components contributed to efficient neuron segmentation.

### 3.3. NeuroSeg-II achieved accurate and generalized neuron segmentation

**FIGURE 6:**
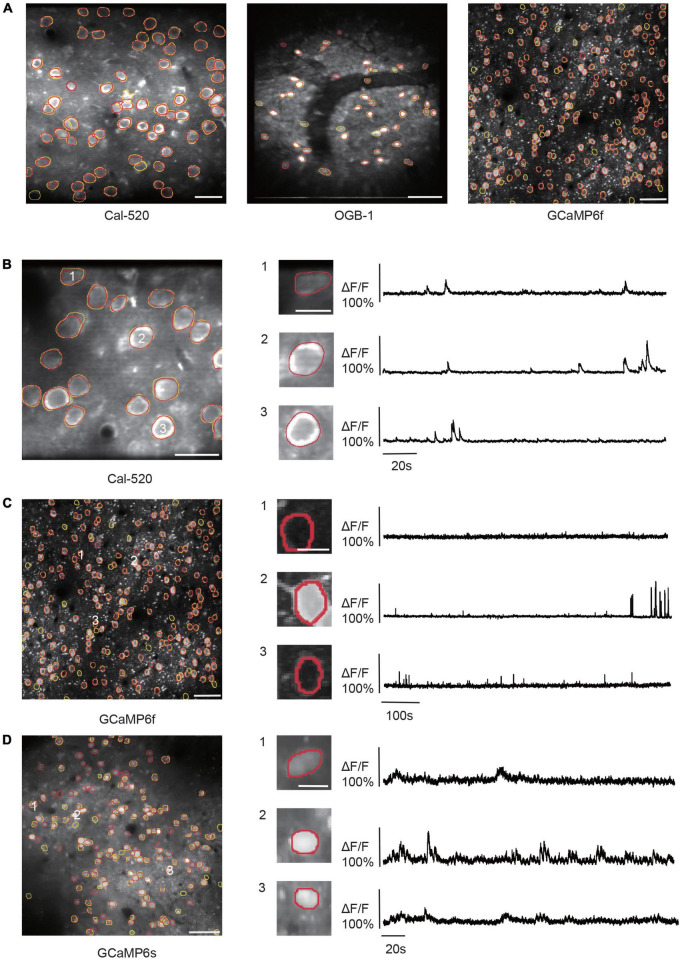
Results of NeuroSeg-II performing generalized neuron segmentation. **(A)** Examples showing the neuron segmentation results for Cal-520 (precision = 0.9677, recall = 0.9375, and F1-score = 0.9524; the dataset from our lab), OGB-1 (precision = 0.875, recall = 0.8333, and F1-score = 0.8536; the dataset from our lab), and GCaMP6f (precision = 0.9779, recall = 0.8634, and F1-score = 0.9207; the dataset from ABO dataset, experiment ID: 536323956). The yellow outlines indicate the GT neurons, and the red outlines indicate the neurons detected by NeuroSeg-II. Cal-520: scale bar, 20 μm. OGB-1: scale bar, 50 μm; GCaMP6f: scale bar, 50 μm. **(B–D)** Examples showing the neuron segmentation of active and inactive neurons in the imaging data for **(B)** Cal-520 (precision = 1.0, recall = 1.0, and F1-score = 1.0; data from our lab), **(C)** GCaMP6f (precision = 0.9779, recall = 0.8634, and F1-score = 0.9171; data from ABO dataset, experiment ID: 536323956), and **(D)** GCaMP6s (precision = 0.6490, recall = 0.7967, and F1-score = 0.7153; data from Neurofinder dataset, experiment ID: 01.01test). The left side is the segmentation result, and the right side is the activities of three representative neurons (scale bar, 10 μm). The yellow outlines indicate the GT neurons, and the red outlines indicate the neurons segmented by NeuroSeg-II. Cal-520: scale bar, 20 μm. GCaMP6f: scale bar, 50 μm. GCaMP6s: scale bar, 50 μm.

Based on the attention mechanism and enhanced feature hierarchy, we trained our NeuroSeg-II with a hybrid dataset and tested it for a generalized neuron segmentation task. As the training dataset contains image features from different Ca^2+^ indicators, imaging scales, neuron activation, brain regions, imaging depths, and labs, NeuroSeg-II performed comprehensive learning and achieved generalized neuron segmentation ability. After hybrid training, we tested two-photon imaging datasets, including three Ca^2+^ indicators (OGB-1, Cal-520, and GCaMP6) and various imaging scales. The results show that the NeuroSeg-II model achieved good performance across three Ca^2+^ indicators and imaging scales (Figure 6A) (Cal-520: F1-score = 0.9524; OGB-1: F1-score = 0.8536; and GCaMP6f: F1-score = 0.9171). In addition, we investigated the activities of the segmented neurons. The results (Figures 6B–D) exhibit that both the active and inactive neurons were recognized by our model, which suggests that the image fusion preprocessing integrates spatiotemporal information and contributes to the improvement of segmentation performance.

**FIGURE 7:**
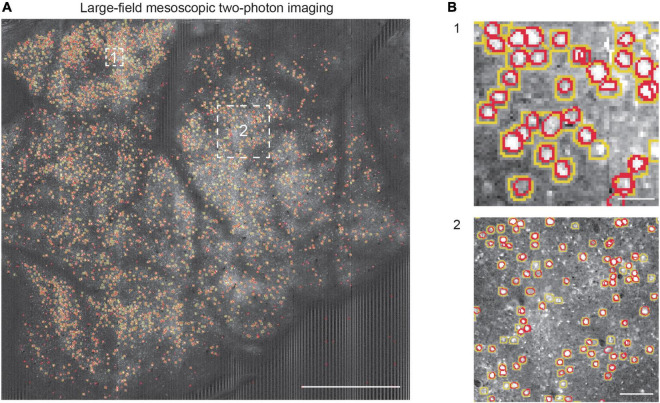
Neuron segmentation in mesoscopic two-photon Ca^2+^ imaging with NeuroSeg-II. **(A)** Segmentation results overlaid on the imaging data of GCaMP6s expressing neurons [mesoscopic data from the study of Sofroniew et al. (2016)]. The yellow outlines indicate the ground truth (GT) neurons, and the red outlines indicate the neurons segmented by NeuroSeg-II. Two regions are highlighted by the white squares and shown at an expanded spatial scale. Scale bar, 1 mm. **(B)** The effect of neuron segmentation at different scales. B1 scale bar, 20 μm. B2 scale bar, 50 μm.

Furthermore, we tested the neuron segmentation capability of the learned NeuroSeg-II model with the large-field mesoscopic two-photon imaging data to demonstrate the generalizability of our trained model, the enhancement of the segmentation effect for small objects, and the dataset advantage of the 2D image processing approach. As the imaging data are too large to deal with the current methods, we first split the data into 306 small images and then recombined the segmentation results from small images to reconstruct the entire FOV. We obtained the final performance by comparing the segmented results with the GT (Figure 7A). We achieved an F1-score of 0.80 (precision: 0.84; recall: 0.76). The result shows that the NeuroSeg-II model obtained by hybrid training could deal with the large-field imaging data with small neurons (Figure 7B), which is challenging for a neuron segmentation task. Moreover, the result also demonstrates that our model maintained reliable performance for untrained images. Therefore, the learned NeuroSeg-II model integrates various neuron characteristics in imaging data and lays a foundation for generalized cell recognition and segmentation.

### 3.4. Comparison of NeuroSeg-II with other neuron segmentation methods

To further validate our proposed network model, we compared NeuroSeg-II with other methods by testing a publicly available two-photon Ca^2+^ imaging dataset (the Neurofinder Challenge dataset). We converted the video data from the Neurofinder dataset into images and used the datasets for training and testing the different methods. Owing to the difference between the image and video for training and testing, we conducted preprocessing and data augmentation on Neurofinder image data to compensate for the information from the videos.

**FIGURE 8:**
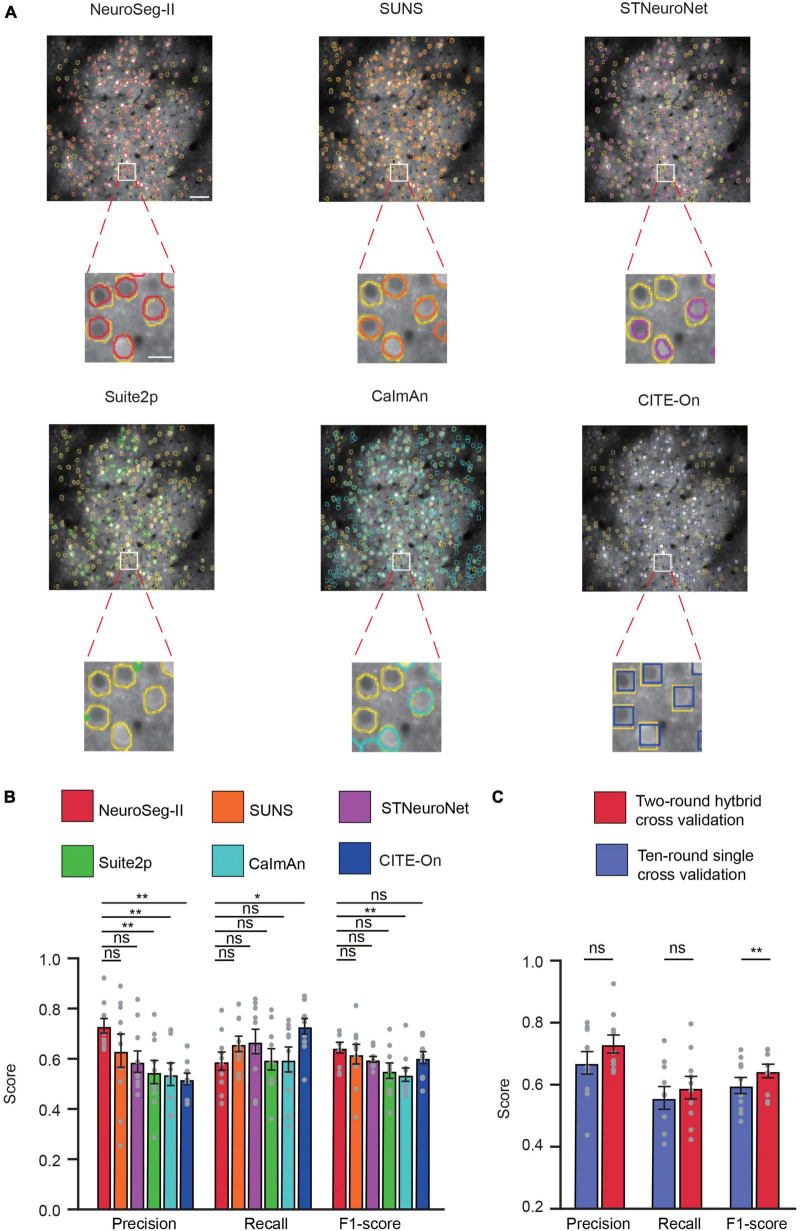
NeuroSeg-II outperformed other neuron segmentation methods in accuracy on the Neurofinder dataset. **(A)** Top: Examples from the Neurofinder dataset (video 02.00) showing the neuron segmentation results of NeuroSeg-II (precision: 0.8272; recall: 0.6054; F1-score: 0.6991), SUNS (precision: 0.7482; recall: 0.6341; F1-score: 0.6865), STNeuroNet (precision: 0.5096; recall: 0.7669; F1-score: 0.6124), Suite2p (precision: 0.7014; recall: 0.3633; F1-score: 0.4787), CaImAn (precision: 0.5301; recall: 0.6978; F1-score: 0.6025), and CITE-On (precision: 0.5191; recall: 0.8395; F1-score: 0.6415), where the segmented neurons are overlaid on the fused image data. Scale bar, 50 μm. The yellow outlines indicate the GT neurons, and the other colors indicate the neurons found by the methods. Bottom: Examples of segmented neurons zoomed in on the white-boxed regions. Scale bar, 5 μm. **(B)** Statistical comparison of NeuroSeg-II with other methods (*P < 0.05, **P < 0.01; *n* = 10 images or videos; ns, not significant; and error bars are SEM). **(C)** Statistical comparison of two-round hybrid cross validation with 10-round single cross validation by NeuroSeg-II (**P < 0.01; *n* = 10 images; ns, not significant; and error bars are SEM). All *P*-values were calculated with a two-sided Wilcoxon signed-rank test. The gray dots represent the scores for each testing image.

Here, we used two-round hybrid cross validation for comparison with other methods. For all other methods, we optimized them using the algorithmic parameters mentioned in the relevant literatures (Pachitariu et al., 2017; Giovannucci et al., 2019; Soltanian-Zadeh et al., 2019; Bao et al., 2021; Sità et al., 2022). The representative image (Figure 8A) with segmented neurons and the GT demonstrate that our network achieved promising performance for this challenging dataset. Based on the 10 videos in the dataset (Figure 8B), NeuroSeg-II achieved higher but statistically insignificant precision (0.731 ± 0.029) than SUNS (0.633 ± 0.067) and STNeuroNet (0.589 ± 0.043), and significantly higher than Suite2p (0.548 ± 0.046) CaImAn (0.539 ± 0.045) and CITE-On (0.519 ± 0.022) (*P* < 0.01, two-sided Wilcoxon signed-rank test, *n* = 10 images). NeuroSeg-II’s recall rate (0.591 ± 0.036) was lower than those of the other methods (SUNS: 0.660 ± 0.031; STNeuroNet: 0.669 ± 0.049; Suite2p: 0.598 ± 0.042; CaImAn: 0.5970 ± 0.049; CITE-On: 0.724 ± 0.030, *P* = 0.0195, two-sided Wilcoxon signed-rank test, *n* = 10 images). NeuroSeg-II’s F1-score (0.644 ± 0.022) was higher, but statistically insignificantly, than those of SUNS (0.619 ± 0.039, *P* = 0.4922), STNeuroNet (0.598 ± 0.012, *P* = 0.1602), Suite2p (0.552 ± 0.031, *P* = 0.0645), and CITE-On (0.602 ± 0.022, *P* = 0.2324), and significantly higher than that of CaImAn (0.537 ± 0.027) (*P* = 0.0059, two-sided Wilcoxon signed-rank test, *n* = 10 images). These comparison results again demonstrate the good generalization capability of NeuroSeg-II, as the high performance was consistent for segmenting neurons in the imaging data acquired from different labs.

In addition, we compared the two-round hybrid cross-validation strategies with a 10-round single cross-validation strategy. The results (Figure 8C) show that the two-round hybrid cross-validation strategy achieved higher performance than the 10-round single cross-validation strategy (precision: *P* = 0.0645; recall: *P* = 0.1309; F1-score: *P* = 0.002; two-sided Wilcoxon signed-rank test, *n* = 10 images). Although the 10-round single cross validation was targeted for the same laboratory image features and labels (Soltanian-Zadeh et al., 2019; Bao et al., 2021), the two-round hybrid cross validation integrated all data information to enrich the image feature for network learning and improve the neuron segmentation performance.

## 4. Discussion

Here, we presented an automated, accurate, and efficient neuron segmentation method for two-photon Ca^2+^ imaging data. The proposed network model was developed based on Mask R-CNN and modified with an attention mechanism and feature hierarchy. We used image fusion preprocessing to integrate spatiotemporal information into 2D images. Our method accurately segments both active and inactive neurons across Ca^2+^ indicators, imaging scales, brain regions, and imaging depths with different experimental setups. Our method was also successfully applied to a large-field mesoscopic image dataset, which is challenging for neuron segmentation. For testing with the Neurofinder dataset, our approach surpassed the performance of the state-of-the-art methods (SUNS, STNeuroNet, Suite2p, CaImAn, and CITE-On) and achieved the highest precision and F1-score.

As the attention mechanism has been reported to be efficient in learning what and where to refine features, we added an ECA-based attention module in our model to focus on neurons properly and thus enhance the feature extraction ability. Guided with the loss function of network, the integrated attention module efficiently helps the whole network by learning which information to emphasize or suppress for distinguishing neurons. In the visualization results (Figure 4D), we saw how the module exactly focused on the targeted neuron regions in a two-photon image. The attention mechanism within a neural network is often used to identify a single and significant object (Hu et al., 2018; Woo et al., 2018; Wang et al., 2019) by emphasizing pivotal features and suppressing background. Our results show that the attention mechanism can also concentrate on multiple objects instead of a single object. The comparison results (Figure 5A) reveal that the ECA module performed well for neuron segmentation. The comparison results indicate that the ECA module had significantly higher accuracy than the network without an attention module, and outperformed the CBAM module. This may be because ECA can achieve more gains for detecting small objects. The ablation study also suggests that using ECA-based attention module produced higher accuracy (Figure 5D), particularly about the precision. These results confirm that the ECA-based network has good generalization ability for neuron segmentation, so the ECA-based attention module makes a significant improvement to our model performance.

To enhance the feature extraction capability, we also modified the model with a path augmentation strategy, including adding an additional down-sampling module and deepening the network structure, and we added “FPN+” path in the network model. The modifications in NeuroSeg-II improve the feature extraction effectively (Figures 5B, C). This strategy enhances the high-level semantic information in the network and enriches various layers of image features in the network. The localization ability of the whole feature hierarchy is further enhanced by combining the activation of the whole object by the high-level network and the activation of the local texture by the low-level network. The receptive field of the feature extraction network is then expanded. Hence, these improvements also contributed to NeuroSeg-II’s good performance. The ablation study also suggests that path augmentation and “FPN+” both contributed the segmentation performance enhancement (Figure 5D), and “FPN+” is particularly useful to improve the recall rate. Some other strategies include (1) augmenting small objects directly to increase the feature information of those objects (Kisantal et al., 2019) and (2) detecting objects on multiscale images to ensure the consistency of scales in ImageNet (Singh et al., 2018). However, these strategies are not suitable for our task owing to the characteristics of neurons in two-photon Ca^2+^ imaging data: (1) The neurons need not be augmented because of the large number in each FOV (the Neurofinder and the ABO datasets contain ∼100–400 neurons); (2) the targeted objects are neurons in images, so there are no different object classes as there are in the general object segmentation task.

The results indicate that NeuroSeg-II enables good segmentation accuracy along with a convenient training and testing process. This approach can avoid misidentification due to out-of-focus fluorescence [termed “neuropil” (Peron et al., 2015)] near neurons. Compared with spatiotemporal methods, our method is also able to use spatiotemporal activity information. The network model with attention mechanism and enhanced feature hierarchy provided accurate neuron segmentation across different datasets and had achieved higher F1-score than spatiotemporal methods, e.g., STNeuroNet. The results of the ablation study confirm that combining components of attention module, path augmentation and “FPN+” provided the best performance, and that is the reason why the proposed network model outperformed the state-of-the-art methods (SUNS, STNeuroNet, Suite2p, CaImAn, and CITE-On). In training by a hybrid imaging dataset, NeuroSeg-II can perform the neuron segmentation task with robustness and generalization ability. NeuroSeg-II was trained with images of various neuron characteristics simultaneously, and then it successfully segmented neurons from multiple datasets, including different Ca^2+^ indicators, brain regions, or depths acquired by independent labs (Figures 5–8). This training strategy also enables NeuroSeg-II to transfer the learned neuron features to new ones, which can quickly and conveniently meet the needs of experimental targeted neurons. The convenience of using NeuroSeg-II is also reflected in the fact that it does not need to adjust the parameters for various types of two-photon Ca^2+^ imaging data. In contrast, the other four methods compared in this paper have specific requirements and adjustments on the parameters of the Neurofinder datasets (Pachitariu et al., 2017; Giovannucci et al., 2019; Soltanian-Zadeh et al., 2019; Bao et al., 2021). As a result, if the dataset is changed and the parameters are not adjusted, the segmentation performance will be degraded. We can make the network learn more neuronal features through the continuous accumulation of datasets and achieve the purpose of rapid training through a small amount of retraining.

Future work should extend the current network to increase processing speed and learning ability for attention-guided multiple sources and small-sample. To achieve accurate and high-speed neuron segmentation, improvements of network architecture (e.g., a light-weight network model) can potentially overcome the tradeoff between accuracy and running speed. It will be helpful to perform fast neuron segmentation and may facilitate large-scale imaging experiments (Fan et al., 2019). For two-photon Ca^2+^ imaging data, the attention mechanism of multiple source domains can extract more image features and reduce image information loss. Small-sample learning can reduce the amount of data required for network learning, improve the training speed, and reduce the time cost of image data processing at the early stage. In addition, using machine learning methods to enhance signal-to-noise ratio of Ca^2+^ imaging data will also reinforce the accuracy of neuron segmentation (Li et al., 2021; Zhuang and Wu, 2022). These methods represent the future development of our work.

## Data availability statement

The imaging data supporting the conclusions of this article are available from the corresponding authors upon reasonable request. The trained NeuroSeg-II model is freely available at https://huggingface.co/XZH-James/NeuroSeg2/tree/main. The code is provided at https://github.com/XZH-James/NeuroSeg2. We used three public datasets to evaluate the performance of neuron segmentation. We used the ABO dataset from https://observatory.brain-map.org/visualcoding/search/overview. We used the Neurofinder dataset from https://github.com/codeneuro/neurofinder and the corresponding labels created from https://github.com/soltanianzadeh/STNeuroNet/tree/master/Markings/Ne urofinder. We used the large-field mesoscopic two-photon imaging dataset from https://github.com/sofroniewn/2pRAM-paper.

## Ethics statement

The two-photon Ca^2 +^ imaging experiment with mice in our lab was approved by the Institutional Animal Care and Use Committee of Third Military Medical University. All experimental procedures were conducted in accordance with animal ethical guidelines of the Third Military Medical University Animal Care and Use Committee.

## Author contributions

XC and XL contributed to the design of the study. JP and MW performed the imaging experiments and acquired the data. ZX, YW, XC, and XL designed the method. ZX, YW, JG, SL, QH, and HJ processed the data sets. XC and XL wrote the manuscript with help from all the other authors. All authors contributed to the article and approved the submitted version.
